# Adolescents Identify Modifiable Community-Level Barriers to Accessing Mental Health and Addiction Services in a Rural Canadian Town: A Survey Study

**DOI:** 10.3390/pediatric16020031

**Published:** 2024-05-06

**Authors:** Hana Marmura, Regina R. F. Cozzi, Heather Blackburn, Oliva Ortiz-Alvarez

**Affiliations:** 1 Faculty of Health Sciences, Western University, London, ON N6A 3K7, Canada; hmarmura@uwo.ca; 2 Biology Department, St. Francis Xavier University, Antigonish, NS B2G 2W5, Canada; rcozzi@stfx.ca; 3 Sexual Violence Prevention and Response Advocate Team, St. Francis Xavier University, Antigonish, NS B2G 2W5, Canada; hblackbu@stfx.ca; 4 Women’s and Children’s Health, Saint Martha’s Regional Hospital, Antigonish, NS B2G 2G4, Canada; 5 Departments of Pediatrics and Family Medicine, Dalhousie University, Halifax, NS B3H 4R2, Canada

**Keywords:** adolescent, mental health, healthcare services, rural medicine, barriers, access

## Abstract

Adolescents are particularly vulnerable to inadequate provision of mental health and addictions care, as services have been traditionally conceptualized to serve the needs of children or adults. Additionally, rural communities have been largely excluded from research investigating mental healthcare access and exhibit unique barriers that warrant targeted interventions. Finally, perspectives from the target population will be most important when understanding how to optimize adolescent mental health and addictions care. Therefore, the purpose of this study was to identify what adolescents in a rural town perceive as barriers to accessing mental health services. We conducted a cross-sectional survey study with high school students to generate ranked lists of the top perceived individual-level, community-level, and overall barriers. A total of 243 high school students responded to the survey. Perceived barriers were predominantly at the community level. Overall, the top barriers reported were a lack of awareness and education regarding mental health, resources, and the nature of treatment. Students who had previously accessed mental health services identified primary barriers related to mental health professionals, whereas students who had not accessed care reported fear and uncertainty as primary barriers. Modifiable community-level factors related to (1) mental health literacy and (2) mental healthcare professionals were identified by adolescents as the main perceived barriers to accessing mental health and addiction services in a rural town. The findings of this preliminary study should inform intervention strategies and further rigorous research for this traditionally underserved target population.

## 1. Introduction

One in ten Canadians over the age of 15 report having experienced at least one mental disorder in the previous 12 months, and about one in five report needing mental health services [1]. Mental health disorders lead to significant health and economic burdens globally and within North America [2]. The significance and impact of mental health disorders on individuals, society, and the healthcare system have been largely underestimated and warrant global action to fund and prioritize relevant treatment and services [2].

Many mental health issues, including mood and psychotic syndromes, start during adolescence, with long-term effects persisting into adulthood [3]. It has been hypothesized that the burden of mental illnesses can be minimized by facilitating timely and effective treatment after symptoms first appear in adolescent-onset conditions [3]. Adolescence is characterized by significant physical and social changes [3], leading to distinct healthcare needs that require individualized interventions. These needs are often not well met within the current mental health services framework, which is conventionally designed for either children or adults [4]. During their transition to adulthood, adolescents develop increasing autonomy and capacity for self-initiated health-seeking behavior. As such, it is critical to include the perspective of adolescents in research regarding their care, rather than only the traditionally studied perspectives of healthcare providers, teachers, and caregivers.

Over the past decade, pediatric emergency room visits have close to doubled in the United States, with the largest rate increase observed in adolescents compared to children or young adults [5]. A concerning number of adolescents present in crisis to the emergency department with signs of severe mental illnesses, having not previously received any mental health services, as evidenced by a five-fold increase in pediatric suicide-related visits to the emergency department [5,6]. This pattern is especially prominent in rural areas [6]. Rural communities face unique challenges in healthcare delivery, often related to a lack of healthcare professionals and exacerbated by a lack of consistent personnel [7]. Rural areas in Canada have been dubbed “communities in crisis” due to a lack of sufficient resources to operate key emergency and ambulatory health services [7]. Within our province of Nova Scotia, youth in rural communities and those with lower socioeconomic status were identified as potentially underserved groups, who may interact with the healthcare system in less cost-effective ways [8].

Therefore, the purpose of this study was to identify the primary barriers to accessing mental health and addictions services, as perceived by adolescents in a rural community. This study was designed as a preliminary descriptive study to investigate an underrepresented group: adolescents in rural communities. This exploratory study is intended to inform potential intervention strategies or policies that can be studied in larger trials.

## 2. Materials and Methods

We conducted a cross-sectional survey study to describe barriers to accessing mental health and addiction services in high school students in the rural town of Antigonish, Nova Scotia, Canada. We adapted a questionnaire originally created by Church and colleagues based on the Canadian Institutes of Health Research’s conceptual model of systems-, community-, and individual barriers to accessing child and youth mental healthcare [9]. In order to tailor the questionnaire to adolescent respondents and increase engagement/completion, we removed questions related to system-level barriers and the identification of mental health conditions. We added questions related to a few additional barriers and facilitators identified in a previous study on family perspectives on pathways to mental healthcare for children and youth in rural communities that we deemed relevant [10]. The final questionnaire was divided into the following sections: perception of individual-level barriers, perception of community-level barriers, and respondent characteristics (demographics, attempts to access mental health services, and awareness of resources). For each proposed barrier, students responded as to whether they strongly disagreed, somewhat disagreed, were neutral, somewhat agreed, or strongly agreed that this was a barrier to accessing mental health and addiction services. A registered nurse and crisis mental health worker and the quality lead in mental health and addictions for the area reviewed the questionnaire to ensure face validity. A summary of the potential barriers asked about are displayed in Table 1, and the full questionnaire is available as Appendix A.

All high school students were eligible to participate in the study. The questionnaire was made available online on a secure platform (Redcap), allowing adolescent participants to respond anonymously. Informed consent was obtained from all participants, implied by questionnaire completion. We distributed the questionnaire from March to June 2019. Ethical approval was obtained from the Nova Scotia Health Authority (NSHA) Ethical Review Board (REB # 1023909). We informed and obtained approval from the relevant stakeholders, including the school board, school staff, and school resource providers, in addition to informing students and parents of the study and its purpose.

A points system was introduced for data analysis, whereby for each barrier, Likert scale responses were allotted points from “strongly agree” (1 point) to “strongly agree” (5 points). The points allotted to each barrier were summed across all respondents and weighted by the number of respondents that answered the question to yield a mean score from 1 to 5. We then generated ranked lists of the top perceived overall-, individual-, and community-level barriers reported by respondents. We completed this analysis for the entire sample and completed a sensitivity analysis, stratifying the sample by students who had and had not previously accessed mental health services. We analyzed the data as collected based on a low percentage of missing data.

A required sample size of 136 respondents was calculated based on a conservative estimate of the prevalence of mental health issues in our population as 10%, a 95% confidence level, and accounting for 15% missing data with a finite population correction for the 700 students who were sent the survey [11].

## 3. Results

### 3.1. Participants

A total of 243 students responded to the survey (response rate: 34%, 234/700), meeting the required sample size. Most respondents were 15 to 16 years old and lived with their parents. About one-third reported that they had tried to access mental health services in the past, and only half of this group were satisfied with the services provided. Most students (>70%) were aware of at least one mental health resource that they were asked about. Descriptive characteristics of the respondents are displayed in Table 2.

### 3.2. Perceived Barriers to Accessing Mental Health Services

**Figure 1:**
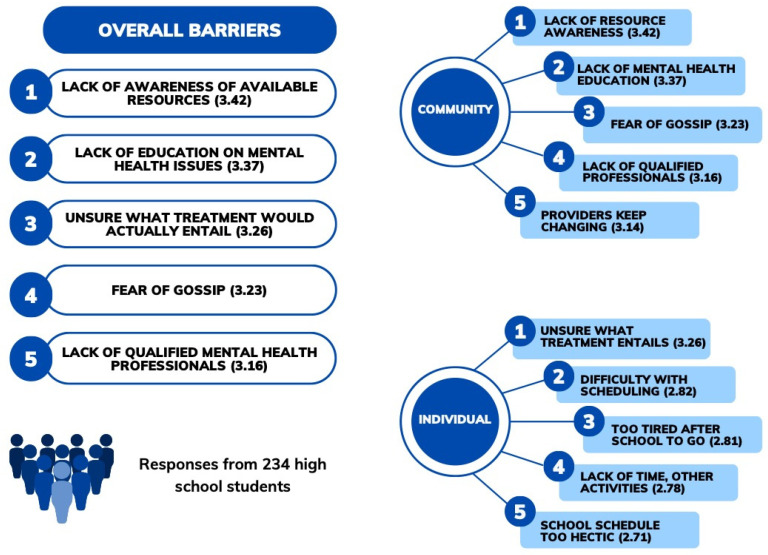
Ranked lists of top perceived overall, community-level, and individual-level barriers to mental health services as reported by 234 high school students in a rural town. Mean scores from Likert scales are indicated in brackets next to the barrier label (range 1 to 5).

The top five perceived overall, community-level, and individual-level barriers to accessing mental health and addiction services across all respondents are shown in Figure 1. When both individual- and community-level barriers were pooled together, community-related factors were more prominent, being listed as four of the top five overall barriers (Figure 1).

The top individual-level barriers identified were similar when respondents were stratified by having previously accessed mental health services, with both groups listing being unsure of what treatment would entail at the top of their list. Importantly, negative past experience(s) with mental health services was listed as the fifth individual-level barrier from those who had accessed services. There were interesting differences amongst the top community-level barriers between those who had and had not accessed services. Both groups shared a lack of education regarding mental health and a lack of awareness of available resources as top five barriers. Students who had previously accessed services reported issues with mental health professionals and services themselves as important barriers, including a lack of qualified mental health professionals, that mental health providers keep changing, and a lack of collaboration/communication among service providers. In contrast, students who had not previously tried to access services perceived fear/social stigmas as important barriers, including a fear of gossip, exclusion, and being judged by friends. The results from the analysis, stratified by previous access to services, are summarized in Table 3.

## 4. Discussion

The main finding of this study is that community-level barriers related to mental health literacy (education, awareness, and knowledge) and mental healthcare providers were identified as the primary barriers to adolescents accessing mental health services in a small rural town. Importantly, community-level barriers are more easily modified by healthcare providers, policy makers, and stakeholders than individual-level barriers and this study has identified feasible targets of interventions, strategies, and policies that are specific to a rural community. We will now discuss the main barriers reported in our study within the context of the wider literature and potential solutions.

A lack of education, awareness, and mental health literacy are the most cited barriers to accessing mental health services [9,10,12]. While our study did not investigate the mechanism of these barriers, poor mental health literacy has been associated with an inability to recognize signs and symptoms of mental health illnesses [9,13]. This lack of knowledge can manifest in individuals as an uncertainty about whether services are needed and how they might help, especially in the uncertain era of adolescence. Ultimately, these barriers likely delay and prevent help-seeking behaviours. Reviews of mental health literacy and education interventions report positive outcomes, including improved knowledge and decreased stigmatizing or negative attitudes around mental health issues [14,15]. Potential education-based interventions could include community-based interventions (targeting the whole community or a specific youth audience), school-based interventions (teaching help-seeking, mental health literacy, and resilience), and training for individuals to better intervene in mental health crises [14,15]. Recommended strategies for developing and implementing successful education interventions include doing preliminary research with the target audience, using a proven theoretical base, tailoring messaging to different groups, using various media, and evaluating the implemented programs [14,15]. The current study represents step one of this process for our community.

A lack of qualified mental health professionals was one of the top five overall barriers reported. Further, respondents who had accessed mental health services expressed negative perceptions of the services received, citing a frequent turnover of mental health service providers and a lack of collaboration/communication amongst providers. A recent systematic review discussed the lack of national institutional cohesion and young-adult-specific policies in Canada [16]. Suggestions to improve the availability and quality of mental health professionals on a policy level are to allocate funding to scale up proven programs and ensure that these services are included in health insurance coverage [16]. Recently, virtual mental health initiatives have become popular as accessible and cost-effective proven models of care [16]. Youth appear willing to consider virtual mental health services, although fewer are willing in a group setting [17]. The advantages of virtual care include improved access to trained professionals and an overcoming of geographical barriers. Adolescents may feel less vulnerable or stigmatized and more comfortable accessing services from their homes and may enjoy the interactive features that technology can provide. For example, Canadian adolescents aged 14–25 who participated in a study investigating the use of an app for mental health services reported liking the increased communication with their provider (via messaging), appointment scheduling, integration of a safety plan, and reminders related to their wellness plan and medications [18]. It is important to note that some youth, especially those in rural areas or from low socioeconomic backgrounds, will face technological barriers that may prevent them from accessing this type of care.

This study is limited by a small sample size and responses from adolescents in a single school, which may limit the generalizability. However, this study aims to serve preliminary research in a specific target audience, to inform interventions that can be investigated in larger studies and may address the mental health literacy and workforce barriers that were clearly identified by adolescents in a rural Canadian town.

## 5. Conclusions

Modifiable community-level factors related to (1) mental health literacy and (2) mental healthcare professionals were identified by adolescents as the main perceived barriers to accessing mental health and addiction services in a rural town. The findings of this preliminary study should inform intervention strategies and further rigorous research for this traditionally underserved target population.

## Figures and Tables

**Table 1:** Summary of individual- and community-level barriers to accessing mental health and addiction services, from the questionnaire distributed to high school students in a rural community.

Individual-Level Barriers	Community-Level Barriers
Lack of transportation Live too far away Parking cost Difficulty with scheduling Lack of time/other activities Too tired after school School schedule too hectic Takes time away from spending time with friends Added stress Health problems or illness Crisis at home Family health problems or illness Family members would prevent or disagree with treatment Unsure of what treatment would entail Previous negative experience with mental health services Embarrassment Fear of being prescribed medication Belief that peoples who access mental health services are “crazy” Belief that accessing mental health services is a sign of weakness Lack of trust in mental health professionals.	Fear of gossip Fear of social exclusion Lack of anonymity Fear of being judged by friends Lack of confidential location/space Fear of shaming my family Same health provider performing multiple roles Lack of collaboration/communication among service providers Mental health providers keep changing Lack of qualified mental health professionals Lack of youth-friendly services Inability of service providers to relate to youth Lack of proper education regarding mental health issues Lack of awareness regarding available resources

Note: For each proposed barrier, students responded as to whether they strongly disagreed, somewhat disagreed, were neutral, somewhat agreed, or strongly agreed that this was a barrier to accessing mental health and addiction services.

**Table 2:** Descriptive characteristics of student respondents.

Characteristic	N (%)
Age	
15–16	159 (65.2)
17–18	66 (27)
19–20	2 (2.5)
Missing	13 (5.3)
Gender	
Male	93 (38.1)
Female	129 (52.9)
Other	7 (2.9)
Prefer Not to Say	4 (1.6)
Missing	11 (4.5)
Living Situation	
With Mom and/or Dad	225 (92.2)
With Other Family Member	5 (2.0)
Foster Home	1 (0.4)
With Friends	4 (1.6)
No Fixed Address	1 (0.4)
Missing	8 (3.3)
Tried to Access Mental Health Services in the Past	
Yes	79 (32.4)
Satisfied	38 (49.4)
Not Satisfied	32 (41.6)
Prefer Not to Say	7 (9.1)
No	154 (63.1)
Prefer Not to Say	5 (2.0)
Missing	6 (2.5)
Awareness of Existing Community Services	
School Plus	173 (73.0)
St. Martha’s Mental Health and Addiction Services	173 (70.9)
Family Services of Eastern Nova Scotia	77 (31.6)
Awareness of Existing Online Resources	
KidsHelpPhone.ca	203 (83.2)
Teenmentalhealth.org	104 (42.6)

**Table 3:** Top five individual-level barriers to mental health and addiction services, stratified by previous access to services.

	Have Accessed Services in the Past	Have Not Accessed Services in the Past/ Prefer Not to Say
	INDIVIDUAL-LEVEL BARRIERS	
**1**	Unsure of what treatment would entail (3.10)	Unsure of what treatment would entail (3.33)
**2**	Too tired after school (3.10)	**Will not have time/other activities (2.83)**
**3**	Scheduling appointment times (2.91)	Scheduling appointment times (2.78)
**4**	School schedule too hectic (2.89)	Too tired after school (2.89)
**5**	**Negative past experience(s) with mental health** **services (2.81)**	School schedule too hectic (2.63)
**COMMUNITY-LEVEL BARRIERS**		
**1**	Lack of education regarding mental health (3.65)	Lack of awareness of available resources (3.42)
**2**	Lack of awareness of available resources (3.40)	**Fear of gossip (3.31)**
**3**	**Lack of qualified mental health professionals (3.23)**	Lack of education regarding mental health (3.23)
**4**	**Mental health providers keep changing (3.21)**	**Fear of exclusion (3.13)**
**5**	**Lack of collaboration/communication among service providers (3.14)**	**Fear of being judged by friends (3.13)**

**Bolded** barriers represent those that differ between groups. Mean scores from Likert scales are indicated in brackets next to barrier description (range 1 to 5).

## Data Availability

Data are available upon request.
